# Cognitive control, cognitive reserve, and memory in the aging bilingual brain

**DOI:** 10.3389/fpsyg.2014.01401

**Published:** 2014-12-03

**Authors:** Angela Grant, Nancy A. Dennis, Ping Li

**Affiliations:** ^1^Department of Psychology, Pennsylvania State University, University ParkPA, USA; ^2^Center for Brain, Behavior and Cognition, Pennsylvania State University, University ParkPA, USA

**Keywords:** aging, bilingualism, brain reserve, cognitive reserve, memory, neuroimaging

## Abstract

In recent years bilingualism has been linked to both advantages in executive control and positive impacts on aging. Such positive cognitive effects of bilingualism have been attributed to the increased need for language control during bilingual processing and increased cognitive reserve, respectively. However, a mechanistic explanation of how bilingual experience contributes to cognitive reserve is still lacking. The current paper proposes a new focus on bilingual memory as an avenue to explore the relationship between executive control and cognitive reserve. We argue that this focus will enhance our understanding of the functional and structural neural mechanisms underlying bilingualism-induced cognitive effects. With this perspective we discuss and integrate recent cognitive and neuroimaging work on bilingual advantage, and suggest an account that links cognitive control, cognitive reserve, and brain reserve in bilingual aging and memory.

Bilingualism^1^ research has recently generated much enthusiasm in the study of the mind and brain (see Diamond, 2010; Bialystok, 2011). What has brought bilingualism to the center stage in cognitive science? One key reason might be the perspective (and the findings) that the bilingual’s experience with two or more languages conveys long-term cognitive benefits. Such benefits are reflected in the fact that, compared to monolinguals, bilinguals typically display enhanced cognitive control abilities, show more mental flexibility, and can better handle tasks involving switching, inhibition, and conflict monitoring (see Bialystok and Barac, 2013 for a review).

In the last decade a large number of studies have been devoted to the study of the cognitive benefits of bilingualism. In these studies, bilinguals generally show smaller effects of conflict (as measured by reduced reaction time) across a variety of executive control tasks, including the Attention Network Test (e.g., Costa et al., 2008; Tao et al., 2011), the Simon task (e.g., Bialystok et al., 2004), and the Stroop task (e.g., Coderre et al., 2013). Additionally, these cognitive advantages have been shown to occur across the lifespan in populations ranging from children (e.g., Bialystok and Feng, 2008) to young adults (e.g., Bialystok et al., 2008) to older adults (e.g., Bialystok et al., 2006). Furthermore, bilinguals, compared with monolinguals, show delayed onset of age-related cognitive decline in varying forms of dementia, such as Alzheimer’s disease, by an impressive average of 4–4.5 years (Bialystok et al., 2007; see also Alladi et al., 2013).

In this paper, we attempt to provide an account of the cognitive effects (and advantages) due to bilingualism from a neurocognitive perspective. Specifically, our goals are to (1) evaluate the behavioral and neural bases of bilingual cognitive advantage, and (2) suggest a mechanistic account for understanding the neural basis of bilingual cognitive reserve in older adults by examining the relationship between executive control, memory, and brain reserve.

A number of researchers have suggested that the bilingual experience in older adults may provide cognitive reserve, a protective mechanism that increases the brain’s ability to cope with pathology (Bialystok et al., 2007; Luk et al., 2012; Abutalebi et al., 2014a,b). In a review of cognitive reserve, Stern (2009) distinguished cognitive reserve from brain reserve. An earlier hypothesis on brain reserve was the threshold hypothesis, according to which there is a critical threshold past which the patient will show clinically marked cognitive deficits (see Satz, 1993). Stern (2009, p. 2016) defined brain reserve as “individual differences in the brain itself allow some people to cope better than others with brain pathology.” In this perspective, the mechanism underlying brain reserve is thought to be quantitative: more neurons, more synapses, better resistance to apoptosis, and so on, are used in the individual to protect against age-related decline. Cognitive reserve, in contrast, was defined by Stern (2009, p. 2016) as “individual differences in how people process tasks allow some to cope better than others with brain pathology.” Bialystok et al. (2007) argue that bilingualism contributes to cognitive reserve by improving the efficiency of executive control processing via extensive, daily, experiences in handling two competing languages (e.g., inhibiting the language not in use; see more discussion below). Although these authors did not formally distinguish cognitive reserve from brain reserve as did Stern (2009), their implicit assumption was that cognitive reserve entails brain reserve (see Luk et al., 2011).

To understand how bilingual experience provides the individual with cognitive reserve, we need to systematically examine the cognition-brain-behavior relationship. On the one hand, there must be a brain basis of cognitive reserve, perhaps reflected as enhanced levels of neuroanatomical changes, for example, increased gray matter density, white matter integrity, and cortical thickness (see Li et al., 2014, for a recent review). This assumption would be consistent with the idea of a cognitive reserve-brain reserve correspondence. On the other hand, there must also be a behavioral basis for cognitive reserve to arise, perhaps due to the increased levels of engagement in tasks or activities that rely on executive control, attention, monitoring, and switching in bilinguals. This paper aims to provide the missing links between cognition, brain, and behavior with regard to the cognitive advantages of bilingualism, specifically focusing on how they can be identified in the aging bilingual brain. We begin by looking at the behavior and cognition correspondence below, and then discuss the neural basis of cognitive reserve.

## BEHAVIORAL BASIS OF THE BILINGUAL COGNITIVE ADVANTAGE

### EXTENT, NATURE, AND TIMING OF BILINGUAL EXPERIENCE

Unlike many cognitive tasks that may be confined to specific skills or specific activities, bilingual language experience is typically intensive, long-term, and practiced on a daily basis with high frequency for most people living in a bilingual environment. It is this intensity or extent of experience to which researchers attribute the advantageous cognitive effects of bilingualism. Recent work from lab training or longitudinal studies has also shown that the extent of bilingual experience often correlates with levels of L2 proficiency and degree of neuroanatomical changes (Mechelli et al., 2004; Grogan et al., 2012; Mårtensson et al., 2012; Schlegel et al., 2012). However, the intensity of experience alone is not sufficient to account for the unique role of bilingualism. Two other factors must also be taken into consideration, the nature of the experience and the timing of it (see Bates, 1999; Li et al., 2014).

In terms of the nature of bilingual experience, it has been suggested that bilingualism can be compared to a “mental juggler” (Kroll and Bialystok, 2013). That is, speaking one language often involves the parallel activation of both of the bilingual’s languages (i.e., non-selective activation of items from both languages; see De Groot, 2013 for a recent review), and therefore, inhibitory control is required to suppress the activation of the non-target language in the bilingual’s language production or recognition process. This active use of inhibitory control in bilingualism has been formalized as hypotheses in the Inhibitory Control model (Green, 1998) and the Bilingual Interactive Activation model (BIA+; Dijkstra and van Heuven, 2002). To effectively produce or understand the current speech stream and avoid constant interference from the unintended language, the bilingual must engage the executive control system to monitor the two languages and inhibit the activation of the other language. This is even more important in a bilingual environment where the speaker has to switch between languages when conversing with speakers of different language backgrounds.

In terms of the timing of bilingual experience, while it is true that in general the earlier a second language (L2) is learned, the more likely native-like proficiency will be obtained [i.e., the age of acquisition (AoA) effect; see Hernandez and Li, 2007], it has now been recognized that very high levels of proficiency in the L2 is possible even when one learns the L2 late in life. Even more encouraging is the evidence that the brain shows considerable malleability as a result of language learning experience, so that both functional and neuroanatomical changes can occur across the lifespan (see Li et al., 2014 for review). Still, there is the question of how the timing of language experience interacts with the intensity or extent and with the nature of the linguistic experience. For example, Abutalebi et al. (2014a) showed that when older bilinguals in the range of 60–80 years of age are examined, the AoA effect may not be that important anymore (i.e., after learning the L2 for 30–50 years, as compared to a few years of difference in the initial age of L2 exposure). This situation differs from younger bilinguals (e.g., college students, our typical experimental subjects) who are in the range of 18–22 years of age, when a few years of AoA difference can be significant.

### DOMAIN-GENERAL COGNITIVE ENHANCEMENT?

Underlying the bilingual cognitive advantage hypothesis is the assumption that experience in one domain (bilingualism) can have a positive impact more broadly across domains (executive control in general cognition). This is because the bilingual language experience engages the executive control system constantly, in some cases a lifetime experience, in the monitoring, selecting, controlling, and switching between multiple languages that may be simultaneously active and competing in the bilingual mind. Such experiences could in turn sharpen the bilingual mind and lead to domain-general, non-linguistic enhancement in information processing (Bialystok and Barac, 2013).

There are several issues that we must resolve before we can completely embrace this domain-general cognitive enhancement argument. First, we must link specific aspects of the bilingual experience with specific components of the executive control system (see Dong and Li, 2014). This includes the study of the similarities and differences between, for example, language switching and non-linguistic task switching. A number of studies have now been devoted to this issue, including the neural substrates of linguistic versus non-linguistic task differences (e.g., Abutalebi et al., 2008; van Heuven et al., 2008; Prior and Gollan, 2011). Second, we need to understand whether language experience is unique in providing domain-general enhancement in executive control, or whether the acquisition of other cognitive tasks or skills can similarly lead to domain-general effects. So far the evidence in this regard has been scanty, although there has been claim that intensive activities such as action video games may enhance resource allocation abilities and hence general cognition (e.g., Bavelier et al., 2012). Related to this is the challenge of how to separate effects due to bilingual experience from effects due to other cognitive activities, as well as from effects of cultural background, language similarity or distance, education, and socio-economic status, among others (see Bialystok and Barac, 2013). Third, there is the issue of whether the cognitive effects of bilingualism are reflected specifically in the enhancement of executive control abilities, or instead to a general cognitive processing advantage. Hilchey and Klein (2011) reviewed a large number of studies and showed that evidence for bilingual advantages in inhibiting, switching or updating is not always reliably found. However, bilinguals consistently show faster RTs overall than do monolinguals (e.g., in both congruent and incongruent trials in the Flanker task), suggesting that it is a general processing advantage that characterizes the cognitive benefits of bilingual experience.

A final issue concerns whether the cognitive advantage of bilingualism depends on the age of the population (see also discussion of AoA effects in Section “Extent, Nature, and Timing of Bilingual Experience”). For example, robust executive control abilities, especially inhibitory control as measured by the Flanker task, have been found with children (e.g., Bialystok et al., 2005; de Abreu et al., 2012) and older adults (Bialystok et al., 2004, 2008), but often not with young adults (Bialystok et al., 2005; Salvatierra and Rosselli, 2011; Paap and Greenberg, 2013). Bialystok and Barac (2013) noted that even when effects of bilingual cognitive advantages are found in young adults, they tend to be more modest in size than those present in children and older adults, and only under certain experimental conditions (e.g., more challenging cognitive tasks; see Costa et al., 2009). In the remainder of this paper, we will focus on examining the neural mechanisms that underlie bilingual cognitive advantages in older adults, in an attempt to understand the relationship between cognitive reserve and brain reserve.

## NEURAL BASIS OF BILINGUAL ADVANTAGE IN THE AGING BRAIN

Given the aforementioned differences in the probability of observing a bilingual advantage in executive control among different age groups, what evidence do we have for the basis of enhanced executive control abilities in aging bilinguals? How does this enhancement provide a protective mechanism, i.e., “cognitive reserve,” against age-related cognitive decline in healthy and abnormal populations? In what follows, we review studies that point to the positive effects of bilingualism on aging, and establish the relationship between cognitive reserve and brain reserve based on extant evidence from the bilingual aging literature.

### COGNITIVE RESERVE, AGE-RELATED DECLINE, AND THE BILINGUAL EFFECT

In a study surveying hospital records of patients, Bialystok et al. (2007) identified a sample of 184 patients who were diagnosed with dementia, most having Alzheimer’s disease. Of these patients, half were monolinguals (91) and half were lifelong bilinguals (93). Their analyses showed that these two groups differed significantly: the bilinguals showed an average delay of 4 years on the age of dementia onset compared with the monolinguals. To see if such a difference might have originated from other potentially confounding factors such as education, the authors examined the patients’ occupational status and found that in fact the monolingual group had more education than the bilingual group, making their results even more striking, as education has previously been observed to delay the onset of dementia (Stern, 2009).

These results were subsequently replicated in two other studies. Craik et al. (2010) collected data from 211 patients who had suffered from Alzheimer’s disease and found that the lifelong bilinguals (102 patients) had experienced symptoms of dementia more than 5 years later than the monolinguals (109 patients). Again, the two groups had equivalent cognitive abilities and the monolinguals had more education than the bilinguals. Similarly, Alladi et al. (2013) found an equivalent advantage for bilinguals and multilinguals over monolinguals in the age of onset of dementia, including Alzheimer’s, frontotemporal, and vascular dementia^2^. Pooling from a large cohort of patients, they found that bilinguals (391) showed symptoms of dementia 4.5 years later than their monolingual (257) counterparts. Moreover, this advantage was not influenced by potential confounding factors including education, sex, occupation, or languages spoken.

In addition to delaying cognitive impairment, bilingualism has also been associated with improving general mental health among aging populations. For example, Bak et al. (2014) recalled 853 participants (262 bilinguals of varied AoA) who had been first tested in 1947 as part of the Lothian Birth Cohort and tested them between 2008 and 2010. Using the data from 1947, they were able to control for childhood intelligence, gender, socioeconomic status, and immigration. Overall, they found that bilingualism provided a protective effect on general intelligence. They also found that multilingualism, as compared to bilingualism, provided even more protection with respect to general intelligence, reading scores, and verbal fluency. Interestingly, the protective effects on general intelligence and reading were identified regardless of age of L2 acquisition, where memory and verbal reasoning were sensitive to age effects. In conclusion, these studies suggest that bilingualism contributes to cognitive reserve in both healthy and disordered aging populations.

### STRUCTURAL NEURAL SUBSTRATES OF COGNITIVE RESERVE

In addition to the data from patient records, studies investigating the effects of bilingualism on cognitive reserve have also used a variety of neuroimaging methodologies. Given that the differences observed spanned several measures of structural integrity, including enhanced gray and white matter as well as enhanced long-range connectivity in bilinguals compared to monolinguals, it has been suggested that the enhanced cognitive and neural functioning in bilinguals may rely upon this enriched neural architecture.

Luk et al. (2011) assessed functional connectivity changes and structural brain changes in aging lifelong bilinguals. Bilinguals, as compared to monolinguals, showed higher white matter integrity in the corpus callosum projecting to the bilateral superior longitudinal fasciculi, the right inferior fronto-occipital fasciculus, and the uncinate fasciculus. In addition, seed-based functional connectivity centered in bilateral inferior frontal gyri showed similar group differences across hemispheres. Specifically, bilinguals exhibited stronger long-range functional connectivity between the frontal cortex and posterior regions including occipital and parietal cortex; whereas monolinguals exhibited greater short-range connectivity mainly centered within the frontal cortex. These results suggest that lifelong bilingual experience may result in enhanced processing and integration of information across disparate brain regions, compared to that found in monolinguals. Highlighting these patterns, the authors suggested that the gray matter atrophy seen in patients with Alzheimer’s disease may be compensated for by increased white matter integrity in bilinguals. Increased white matter integrity is an example of brain reserve, and in this case may form the neural basis for the foundation of bilingual cognitive reserve.

Abutalebi et al. (2014a) recently observed another form of brain reserve in bilinguals. They compared gray matter volume (GMV) of aging monolinguals and late bilinguals. Their analysis found that although both groups showed effects of aging, bilinguals showed significantly higher GMV in the left temporal pole (i.e., the anterior portion of the left inferior temporal gyrus). In addition, bilinguals showed higher GMV in the right temporal pole and bilateral orbitofrontal cortex compared with monolinguals. However, only the difference in the left temporal pole was significantly predicted by performance on an L2 naming task. The authors argue that the demands exerted by bilingual language processing affect not only frontal control regions, but also more posterior (temporal and parietal) areas associated with semantic processing. Corroborating this argument, Abutalebi et al. (2014b) showed that Cantonese–English and Cantonese–Mandarin bilinguals, compared with monolingual controls, had increased GMV in the left and right inferior parietal lobule (IPL). The IPL has been implicated in lexical representation, semantic integration, and phonological working memory, and increased GMV in this region could be attributed to the bilingual’s experience with a large new vocabulary in the L2 (Mechelli et al., 2004; Richardson and Price, 2009; Della Rosa et al., 2013; see Li et al., 2014 for reviews).

In contrast with the results observed by Luk et al. (2011), Gold et al. (2013a), and Abutalebi et al. (2014a,b) found that bilinguals exhibited cognitive, rather than brain, reserve. That is, in their DTI and VBM analyses of 20 healthy lifelong bilinguals and 63 healthy monolinguals, they found that the two groups showed no difference in GMV, and that the bilinguals actually showed decreased levels of white matter integrity (as measured by fractional anisotropy and radial diffusivity) than the monolinguals. Despite these structural deficits, the bilinguals were able to perform equivalently to the monolinguals on a range of tasks, including IQ, working and episodic memory, and task switching. This pattern of results precisely fits the definition of cognitive reserve: equal performance in the face of structural pathology (Stern, 2009). The authors interpret these results as being potentially due to a higher incidence of pre-clinical Alzheimer’s disease within their sample, especially given that they observed deficits in white matter tracts (specifically within the inferior longitudinal fasciculus/inferior fronto-occipital fasciculus) commonly affected by Alzheimer’s disease. Interestingly, the authors note that the tracts associated with the executive control network ranging from the lateral frontal cortex back to the parietal cortex (e.g., the superior longitudinal fasciculus and anterior limb of the internal capsule) were largely preserved in their bilingual group.

Further support for this link between bilingualism and cognitive reserve comes from the only study that examined functional differences between aging bilinguals and monolinguals (Gold et al., 2013b). They found that older bilinguals (the same sample from Gold et al., 2013a) showed less activity in several frontal regions in association with smaller proportional switch costs compared to age-matched monolinguals, although both groups showed more frontal activation than the younger comparison groups. The combination of reduced neural recruitment and improved cognitive performance is often characterized in the aging field as reflecting greater neural efficiency in high vs. low performing individuals (Rypma et al., 2005, 2006; Grady, 2008).

To review, the structural and functional imaging results, consistent with previous patient data reviewed so far, suggest that brain differences between bilinguals and monolinguals may underlie group differences associated with enhanced cognitive functioning in older bilingual, as compared with monolingual, adults. These structural and functional effects extend across the language and executive control networks, which overlap in keys areas including the prefrontal cortex (PFC), IPL, anterior cingulate cortex, and basal ganglia (Hervais-Adelman et al., 2011). Importantly, the neural networks associated with bilingualism overlap considerably with the neural networks that commonly decline in aging (see discussion below on the overlap between memory and language systems). Such extensive consequences of bilingual experience on the brain’s anatomical architecture and cognitive processing are both unique and important in that they reflect the results of a lifelong experiential context juggling multiple languages, a process that involves both language control and executive control. Compared with most other training experiences that typically produce positive results in a single domain, the bilingual experience seems to have far-reaching consequences for the mind and the brain (Dye et al., 2009; Kroll and Bialystok, 2013).

## BILINGUAL COGNITIVE ADVANTAGES: PERSPECTIVES FROM MEMORY AND AGING

Given the extent to which the bilingual experience appears to affect the brain as discussed in Section “Neural Basis of Bilingual Advantage in the Aging Brain,” it is surprising that bilingual memory performance remains relatively understudied to date. It is even more surprising when one considers the amount of media attention that research on the protective effects of bilingualism has received, especially the studies on Alzheimer’s disease, a disease that primarily affects memory (Dubois et al., 2007). In addition, studies of memory, especially episodic memory and retrieval, have shown that recollection commonly elicits activity not only in the medial temporal lobe (MTL), but also the frontal executive systems and the sensory areas associated with the memory being retrieved, suggesting considerable overlap between the areas associated with memory and those associated with bilingual brain reserve (Dobbins and Davachi, 2006). This link is further supported by the existence of overlapping brain regions involved in both memory and language more generally. For example, Rodríguez-Fornells et al. (2009) hypothesized that the MTL is critical for initial word learning in the second language, and data from other studies are consistent with this hypothesis. Mårtensson et al. (2012) found that hippocampal volume increased after intensive training in simultaneous interpreting, as did cortical thickness of the left PFC (specifically the inferior frontal gyrus), a region that has also been implicated in encoding success (Rizio and Dennis, 2013). In addition, Stein et al. (2012) and Hosoda et al. (2013) both found increases of gray matter density in the left anterior temporal lobe (ATL), an area critical for verbal memory (Bonelli et al., 2013), and the increases positively correlated with the learner’s proficiency level in the L2. The close language-memory relationship has led some researchers to propose that foreign language learning should be explored as a potential treatment to build cognitive reserve in the elderly (e.g., Antoniou et al., 2013). It is also critical, however, to examine the relationship between bilingualism and memory systems in the current bilingual elderly population, as memory research in older adults has the potential to help us understand not only the bilingual advantage as it applies to healthy aging, but also to aging-related cognitive declines.

### THE POSTERIOR-TO-ANTERIOR SHIFT IN AGING (PASA) HYPOTHESIS

**FIGURE 1:**
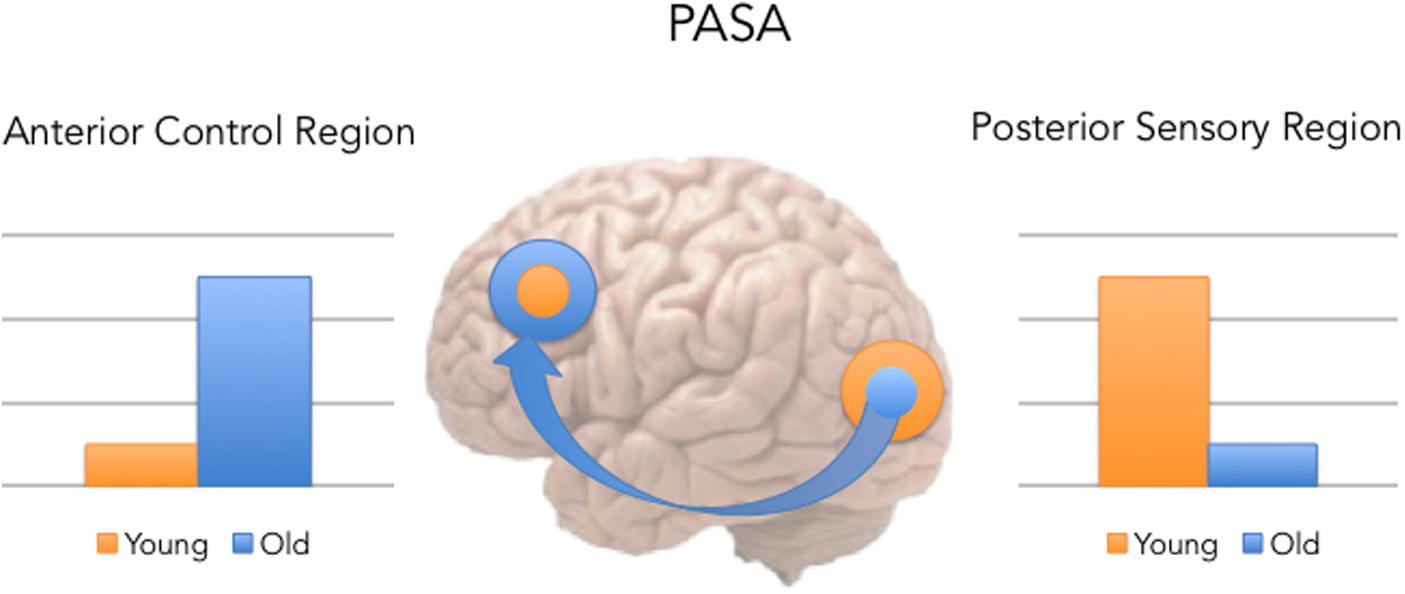
**Posterior-to-anterior shift in aging (PASA) predicts that with age comes a shift, such that older participants show greater activity in the left dorsolateral prefrontal cortex (DLPFC) and less activity in the left visual cortex during memory tasks, while younger adults show the reverse pattern**.

One specific hypothesis that aids us in making the link between memory and bilingualism is posterior-to-anterior shift in aging (PASA), a hypothesis based on functional neuroimaging studies of memory and aging (see **Figure 1** below; for a review see Dennis and Cabeza, 2008).

The PASA pattern of results has been observed across many cognitive tasks in healthy aging (see review in Dennis and Cabeza, 2008). To understand the consequences of the PASA, we need to understand that recollection is commonly associated with activity in frontal executive systems and the sensory areas associated with that experience (e.g., fusiform gyrus activity for the memory of a face), as well as the MTL (Dobbins and Davachi, 2006). PASA’s basic hypothesis is that in normal healthy aging, at least for the monolingual speakers, older adults, compared to younger adults, exhibit age-related decreases in neural activity in posterior sensory areas of the cortex, while at the same time show increases in PFC [especially dorsolateral prefrontal cortex (DLPFC)] activity. PASA is generally considered a form of neural compensation in aging, given that this increased reliance on frontal instead of posterior cortical regions allows the older adults to preserve cognitive performance at a level comparable to younger adults.

Within the aging population, enhanced functioning in bilinguals (compared to monolinguals) is consistent with the PASA hypothesis. Previous research (e.g., Ossher et al., 2013) has focused on the role of bilingualism in enhancing the frontal systems involved in executive function, thus allowing for greater cognitive reserve in bilingualism and memory. Although the frontal activity observed in PASA is characterized as compensatory for reduced activation in the posterior brain regions, the bilingual aging studies reviewed so far suggest that bilingual experience serves as another compensatory mechanism to help mitigate age-related declines by not only engaging the frontal systems, but also preserving structures in posterior areas of the cortex and their connections with the frontal cortex. That is, although monolingual older adults experience the posterior to anterior shifting, in bilingual aging adults, the posterior regions have not declined as substantially, and hence bilinguals show less of the actual shifting in the PASA patterns as in monolinguals. Recent results from Abutalebi et al. (2014a,b) support this preservation hypothesis, with an emphasis on the temporal and parietal regions. As mentioned earlier, Abutalebi et al. (2014a,b) found that aging bilinguals showed enhanced GMV compared to aging monolinguals in the left ATL and in the IPL. Moreover, unlike monolinguals who rely mainly on the frontal cortex to compensate for performance compromised by aging, bilinguals also preserve the frontal-posterior connectivity in addition to the functioning of the posterior regions. For example, Luk et al. (2011) found that aging monolinguals exhibited connectivity patterns congruent with PASA at rest, but aging bilinguals showed greater connectivity between the IFG and posterior areas including the middle temporal and occipital gyri, precuneus, the right IPL, and the caudate.

### ROLE OF THE PFC AND FRONTAL-POSTERIOR AND FRONTAL-BASAL GANGLIA CONNECTIVITY

**FIGURE 2:**
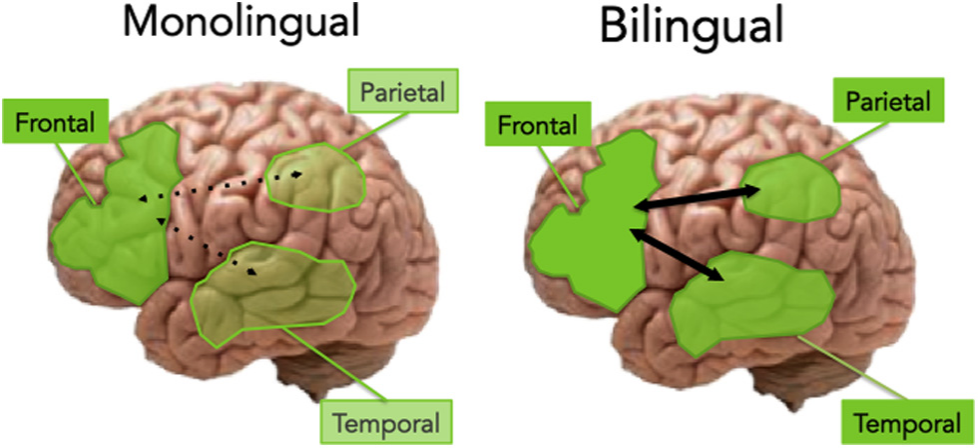
**An illustration of the monolingual vs. bilingual aging brain**. In monolinguals, aging is associated with an increased reliance on the frontal regions, according to the PASA hypothesis. In bilinguals, the aging brain shows preservation of the posterior regions (including temporal and parietal cortex), as well as increased connectivity between frontal and posterior areas, leading to cognitive reserve.

The extant evidence so far suggests that aging bilinguals exhibit preserved GMV in the posterior regions (in particular the temporal and parietal regions) and enhanced connectivity between the PFC and posterior regions (Luk et al., 2011; Abutalebi et al., 2014a,b). Given this evidence, we propose that the enhancement of PFC function, along with preserved temporal cortex and increased frontal-posterior connectivity may underlie the brain reserve of bilingualism. **Figure 2** illustrates this proposal.

**FIGURE 3:**
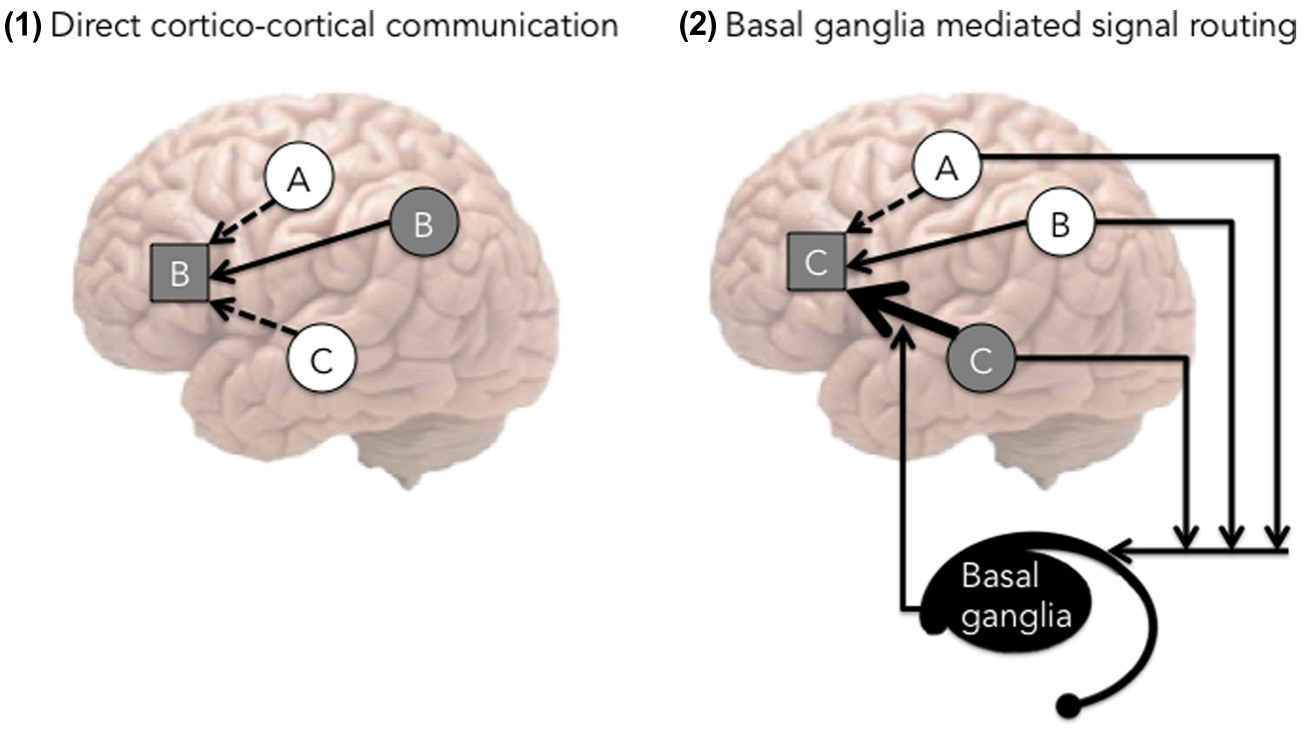
**The conditional routing model, adapted from Stocco et al. (2014).** In this figure, **Side 1** represents a prototypical situation, where prefrontal cortex (PFC) is receiving multiple concurrent signals. In this situation, the strongest concurrent signal (from region B) is most likely to affect the PFC (hence the figure shows the impact of connection B in the prefrontal cortex). **Side 2** exemplifies the role of the basal ganglia, which serves as a mechanism to modify cortical pathways. In this case, the basal ganglia strengthen the signal particularly from area C (hence the figure features the impact of connection C).

Our proposal is consistent with a recent model by Stocco et al. (2014) that describes how the executive control advantage may develop in the framework of the conditional routing model. According to Stocco et al. (2014), the basal ganglia act to increase the strength of the cortical connections that would otherwise have not been selected due to lower resting activation (see **Figure 3**). Stocco et al. (2014) argued that language switching is a process analogous to picking the connection with lower resting activation. Because bilingualism by necessity involves language switching, the authors suggested that bilingualism improves top–down control ability, which includes both language control and general cognitive control. This model is congruent with a number of neuroimaging studies implicating the basal ganglia and specifically the caudate nucleus as being critical for language and cognitive control (e.g., Crinion et al., 2006; Tan et al., 2011), as well as a recent neuropsychological study that found direct evidence via intra-operative electrical stimulation of the role of the left caudate in both language and cognitive control (Wang et al., 2013). To summarize, the conditional routing model suggests that the role of the basal ganglia is to strengthen previously established connections in order to over-ride the currently most active connection, as is necessary during language or task switching.

As we suggested earlier, these benefits should also support memory performance. Specifically, enhancement of frontal regions should allow for the preservation of memory skills involving executive control, such as recollection, while brain reserve in posterior cortical regions and improved long-range connectivity may enhance older bilinguals’ ability to encode and retrieve details of past events, a function that is typically impaired in healthy aging (e.g., Park et al., 2004; Dennis et al., 2007; Gutchess et al., 2007). While monolinguals compensate for reduced long-range connectivity with increased short-range connectivity and activity within the frontal cortex (i.e., PASA; Dennis and Cabeza, 2008), the research that we have reviewed so far suggests that aging bilinguals compensate less drastically in this manner than aging monolinguals (Luk et al., 2011; Gold et al., 2013a). We suggest that this reduced PASA shift in bilinguals is due to their enhancements in the posterior regions (e.g., the medial temporal regions, the temporal pole, and inferior parietal cortex), as well as the connectivity between these areas with the PFC and with the basal ganglia (Stocco et al., 2014).

### THE ROLE OF EXECUTIVE CONTROL AND BRAIN RESERVE IN EPISODIC MEMORY RETRIEVAL

Examined against the above proposal, brain reserve resulting from bilingualism not only supports executive control tasks, but memory functioning as well. Although there are as yet no neuroimaging studies investigating this relationship, a small, but growing, literature has examined memory performance in older bilinguals at the behavioral level. Results from these studies have the potential to help us understand the nature of bilingual brain reserve and its relationship to the incidence and progression of dementia.

One of the first studies in this area by Wodniecka et al. (2010) found that older bilinguals are advantaged at particularly difficult recollection memory tasks compared to older monolinguals, although younger participants (monolingual and bilingual) outperformed both older groups. At the same time, there was no difference between older monolinguals and bilinguals’ ability to remember familiar items. This distinction is important because recollection memory, in particular, requires the use of executive control to select the specific details of the memory over the gist content, whereas familiarity is characterized by an inability to remember details despite a feeling that the item has been seen before. These results consequently suggest that the bilingual executive control advantage does indeed extend to memory, as they found that bilinguals were selectively advantaged in recollection as opposed to familiarity judgments.

Further evidence for the role of bilingualism on memory performance comes from another study by Ljungberg et al. (2013). Similar to Bak et al.’s (2014) longitudinal study that found preserved memory performance among early bilinguals (see Cognitive Reserve, Age-Related Decline, and the Bilingual Effect), Ljungberg et al. (2013) presented a compelling case for a bilingual advantage in episodic memory in the form of a 20-year longitudinal study. Participants were primarily late bilinguals matched on nationality, gender, education, and general intelligence, ranging from ages 35 to 70 at the first testing session. Their data was drawn from a larger study, the Betula Prospective Cohort Study, and included performance on three types of recall tasks, as well as letter and category fluency. Ljungberg et al. (2013) found that bilinguals were significantly advantaged on the episodic recall tasks, and that this advantage did not interact with age. A similar advantage was found for letter, but not category, fluency. While category fluency allows for one to select items relatively freely from one’s existing semantic network, letter fluency requires that one inhibit semantic and occasionally phonological connections in order to recall other words that fit the orthographic criterion, and consequently can be interpreted as an executive control task (see Martin et al., 1994). In short, Ljungberg et al. (2013) found an advantage for bilinguals in episodic recall that co-occurred with increased performance on another executive control task.

In a recent study, Schroeder and Marian (2012) further replicated the pattern of results from the above two studies that shows benefits of bilingualism for recollection memory under conditions requiring executive control. They found that older bilinguals (AoA of approximately 15) performed better than older monolinguals on an episodic memory retrieval task. This task required them to remember specific aspects of scenes, which requires executive control via the inhibition of the gist of the scene. Critically, there was also a correlation between the results on that task and the Simon task, a common measure of executive control. Specifically, the bilinguals’ accuracy on the Simon task was positively correlated with the number of scenes recalled. The monolinguals showed a similar correlation, but the correlation was non-significant. Schroeder and Marian (2012) interpreted these results to suggest that bilinguals’ better performance on the episodic memory task was due to improved executive control abilities. The authors went on to suggest that bilinguals’ “extra reliance on the MTL memory system may exercise and enhance its functioning” (p. 9), allowing for better recall. Although Schroeder and Marian (2012) did not have neuro-imaging data to support this idea, there is support for the hypothesis that the neural basis of the bilingual advantage might involve the temporal and parietal cortex, as has been discussed.

## CONCLUSION AND FUTURE DIRECTIONS

The studies of bilingual memory to date appear to suggest that bilinguals do show an advantage for aspects of memory that require executive control, such as episodic and verbal recall. As discussed in Section “Bilingual Cognitive Advantages: Perspectives from Memory and Aging,” we propose that this advantage may stem from two complimentary mechanisms, a frontal advantage and an advantage in making long-range connections between the PFC and posterior areas of the cortex, which are critical for successful recollection (Dobbins and Davachi, 2006). As illustrated in **Figure 2**, these two mechanisms work differently in monolinguals versus bilinguals, due to their differential experience with language control. As language selection and retrieval occur not only in the frontal cortex, but also in the temporal and parietal cortex (e.g., Abutalebi et al., 2008, 2014a,b), we expect to see strengthening of both frontal and temporal cortical pathways as a result of bilingualism. Current theories of semantic memory have implicated the temporal and inferior parietal cortex as convergence zones for the storage of representations (for a review, see Binder and Desai, 2011), and the understanding of the connectivity between these regions and the executive control system present an exciting opportunity for new research in bilingualism.

Thus, the studies of aging and memory in monolinguals compared with bilinguals provide a bridge for understanding the bilingual advantage in executive control and the cognitive reserve against Alzheimer’s dementia that has been observed in bilingual populations (e.g., Craik et al., 2010; Alladi et al., 2013). On the one hand, the enhanced executive control ability provides the basis for increased cognitive reserve, and on the other, cognitive reserve is sub-served by brain reserve in terms of increased neuroanatomical integrity or density. The analyses presented in this paper only represent an initial attempt at identifying the relationships among bilingual language experience, executive control, episodic memory, and the mechanisms that support the bilingual advantage through cognitive reserve and brain reserve.

Our analyses so far suggest that bilinguals’ cognitive and brain reserves share the same mechanism as their advantaged executive control processing, for example, in the bilinguals’ use of the basal ganglia to strengthen weaker cortical circuits (Stocco et al., 2014). Such strengthening leads to functional and structural consequences, such as increased white matter and gray matter. These structural changes are suggestive of synaptogenesis and dendritic morphology (in the case of gray matter) and increased myelination (in the case of white matter), which allows for more efficient signal communication (see Antoniou et al., 2013; García-Pentón et al., 2014; Li et al., 2014). The large scale of the networks associated with bilingualism makes such improvements critical to allow for fluent speech, and the overlapping nature of this network (between bilingualism and the executive control network) makes it likely that such benefits due to bilingualism would translate into both executive control benefits and consequently, brain reserve for aging-related memory decline.

Future research is needed to elucidate the mechanisms of bilingualism and its cognitive and neural substrates. To understand the implications of functional connectivity between frontal cortex and posterior cortical areas on memory, we need to conduct studies examining functional connectivity in younger and older bilinguals while they complete memory tasks. Such experiments would potentially allow for the differentiation and specification of the role of each of the types of reserve due to bilingualism, that is, structural connectivity and volume in the executive function network. Our general prediction is that like healthy monolinguals, aging bilinguals will exhibit a PASA pattern of neural recruitment compared to young adults during memory and other cognitive tasks. Future experiments comparing young and aging bilinguals, as opposed to only aging monolinguals and bilinguals, will be critical to establish such a parallel.

Another important direction would to be conduct longitudinal research to track the use and development of multiple languages. As we mentioned in Section “Extent, Nature, and Timing of Bilingual Experience,” acquiring a second language appears to have profound structural impacts on the brain (e.g., Mårtensson et al., 2012; Della Rosa et al., 2013; see Li et al., 2014 for review). However, the only longitudinal study of memory performance in bilinguals to date (Ljungberg et al., 2013) has focused on behavioral performance, as reviewed above. Consequently, we do not yet know how the bilingual brain changes over time on tasks that tap into long-term or working memory. Understanding such changes in healthy aging bilinguals and monolinguals and the differences between them will help us to better understand not only cognitive reserve, but also abnormal aging in general.

A final promising line of research could be the study of the effects of foreign language training on memory in older adults. Antoniou et al. (2013) recently called for such a training program in order to explicate the role of bilingualism in cognitive reserve, especially relating to executive control tasks. As we have suggested in this paper, however, executive control appears to have a significant influence on memory as well, and given the results suggesting that older adults also show gray matter changes in response to training (Lövdén et al., 2013) we agree that such a study could be extremely informative not only regarding the cognitive reserve of executive control abilities, but also of memory. If, as we predict, such a study would show either enhanced or preserved memory abilities, as well as executive control abilities, the implication would be that the findings of bilingual cognitive reserve in the face of dementia are in fact related to enhancements of the cognitive control system.

In conclusion, research in the junction of bilingualism, memory, and cognitive and brain reserve can provide significant insights into current debates on bilingual cognitive advantages. Based on the current neuroimaging evidence concerning the bilingual advantage in older adults and on models of aging and memory, we suggest examining brain reserve in not just the frontal cortex, but also its connectivity with the temporal, parietal, and subcortical areas, and how these neural correlates underlie the cognitive reserve in older bilinguals to protect against age-related cognitive declines.

## Conflict of Interest Statement

The authors declare that the research was conducted in the absence of any commercial or financial relationships that could be construed as a potential conflict of interest.

## Footnotes

1 We use the terms “bilingualism” or “bilingual” here inclusively to refer to both bilingual (two languages) and multilingual (more than two languages) situations.

2 But see Chertkow et al. (2010) for evidence of a multilingual but not bilingual advantage, and Zahodne et al. (2014) for a discussion of a null result.
